# Phase I/II trial of local interstitial chemotherapy with arsenic trioxide in patients with newly diagnosed glioma

**DOI:** 10.3389/fneur.2022.1001829

**Published:** 2022-08-30

**Authors:** Dayong Han, Lei Teng, Xiaoxiong Wang, Yunbo Zhen, Xiaofeng Chen, Mingchun Yang, Ming Gao, Guang Yang, Mingyang Han, Ligang Wang, Jiajun Xu, Yue Li, Alina Shumadalova, Shiguang Zhao

**Affiliations:** ^1^Department of Neurosurgery, The First Affiliated Hospital of Harbin Medical University, Harbin, China; ^2^Institute of Brain Science, Harbin Medical University, Harbin, China; ^3^Institute of Neuroscience, Sino-Russian Medical Research Center, Harbin Medical University, Harbin, China; ^4^Department of Neurosurgery, Shenzhen University General Hospital, Shenzhen, China; ^5^Department of General Chemistry, Bashkir State Medical University, Ufa, Russia

**Keywords:** glioma, arsenic trioxide, interstitial chemotherapy, clinical trial, toxicity

## Abstract

**Background:**

Glioma is the most common primary brain tumor in adults with poor prognosis. The glioma patients benefit from STUPP strategy, including maximum and safe resection and adjuvant radiotherapy and chemotherapy. Arsenic trioxide could inhibit various tumors. However, it is a challenge to evaluate the efficiency and safety of srsenic trioxide in glioma patients.

**Objective:**

The arsenic trioxide has the potent therapeutic effect on glioma. However, the safety and efficacy of local interstitial chemotherapy with arsenic trioxide in newly diagnosed glioma patients is unclear.

**Methods:**

All patients received partial or complete tumor resection and intraoperative implantation of Ommaya reservoirs followed by standard radiotherapy. Arsenic trioxide with the starting dose 0.3 mg was administered *via* an Ommaya reservoir catheter inserted into the tumor cavity for 5 consecutive days every 3 months for a total of eight cycles unless tumor progression or excessive toxicity was observed.

**Results:**

No hematological or grade 4 non-hematological toxicity was observed in any patient during arsenic trioxide treatment. The maximum tolerated dose of 1.5 mg of arsenic trioxide was safe and well tolerated. The median overall survival for WHO grade 3 glioma was 33.6 months, and for glioblastoma was 13.9 months. The median progression-free survival for WHO grade 2 glioma was 40.3 months, for grade 3 glioma was 21.5 months, and for glioblastoma was 9.5 months.

**Conclusion:**

These results suggest that arsenic trioxide is safe and well tolerated with local delivery into the tumor cavity of the brain, and the dose recommended for a phase II trial is 1.5 mg.

## Introduction

Glioma is the most common primary brain tumor in adults and is a seldom curable tumor (1, 2). The prognosis for patients with high-grade glioma remains very poor. Treatment for patients with glioma includes surgical resection followed by radiation therapy and chemotherapy (3). The commonly used chemotherapeutic agents in patients with glioma include temozolomide (TMZ), cilengitide, procarbazine, lomustine and vincristine. Survival benefits from adjuvant chemotherapy have been demonstrated in several clinical trials (4–8). Those benefits have been confirmed by an EORTC/NCIC clinical trial in which TMZ was efficacious for patients with GBM as part of first-line therapy with concomitant and adjuvant oral chemotherapeutics. Despite aggressive multimodal therapy, patients with glioblastoma (GBM) have a poor prognosis with a median survival of approximately 12–15 months (7, 9, 10).

Arsenic trioxide (ATO) is an FDA-approved drug and has demonstrated efficacy for the treatment of acute promyelocytic leukemia (11–13). ATO can also induce apoptosis in a wide variety of solid tumors (14–20). Our previous studies have demonstrated its potent therapeutic effects on glioma *in vitro* and *in vivo* (21–23). The safety of ATO therapy *via* traditional drug delivery has been validated in pediatric and adult patients with glioma in a phase I study (24, 25). However, the blood-brain barrier (BBB) is a major obstacle in the use of chemotherapy to treat glioma, and traditional drug delivery at therapeutic doses of chemotherapeutic agents is often accompanied by severe, systemic cytotoxic effects. Direct administration of chemotherapeutic reagents *via* an Ommaya reservoir could theoretically bypass the BBB and directly deliver reagents into the brain tumor (16, 26). This approach may increase the concentration of chemotherapeutic reagents at the tumor site and decrease systemic toxicity.

This study was undertaken to increase the concentration of ATO at the site of glioma, reduce the systemic toxicity of ATO, and evaluate the maximum tolerated dose (MTD) and the treatment effect of ATO on glioma. ATO was locally delivered into a surgically created cavity *via* an Ommaya reservoir in patients with different pathological grades of glioma after receiving surgery and radiotherapy (RT). Thus, this is the first phase I/II trial to investigate the safety and efficacy of ATO locally delivered in patients with newly diagnosed glioma.

## Materials and methods

### Study design

This single-center, open-label, phase I/II trial investigated the safety and efficacy of ATO as a single agent in patients with newly diagnosed glioma. All patients received ATO in the neurosurgical department of The First Affiliated Hospital of Harbin Medical University from February 2004 to December 2007 after they had provided written informed consent. This study was approved by the ethics committees of the First Affiliated Hospital of Harbin Medical University.

A dose escalation study with modified Fibonacci series design was performed to determine the MTD of ATO. Successive cohorts of patients with WHO grade 2 to 3 glioma were enrolled in the dose escalation study because they may have longer survival times than those of patients with GBM. All patients enrolled were evaluated for the toxicity and efficacy of ATO. An initial dose of 0.3 mg was based on previous animal and human studies (13, 27, 28). Three patients were enrolled in each of the initial (0.3 mg) and second (0.6 mg) dose levels. Six patients were enrolled at the third dose level of 1.0 mg, which was close to the expected MTD. Escalation occurred in the same patient over several doses. Patients were enrolled at every dose level according to their hospital admission order. The injected dose of ATO was maintained for every cycle of 5 consecutive days of treatment in the phase I trial. If one-third of the patients experienced dose-limiting toxicity (DLT), the next cohort of the same number of patients was enrolled at the same dose level. MTD was confirmed by two positive results if one-third of the patients experienced DLT. Dose reduction was allowed upon the occurrence of a DLT after treatment. Thus, the previous dose level was defined as the MTD for a phase II study. The primary end point was confirmation of the MTD. The secondary end points were overall survival (OS) and progression-free survival (PFS).

### Patient selection

**Figure 1:**
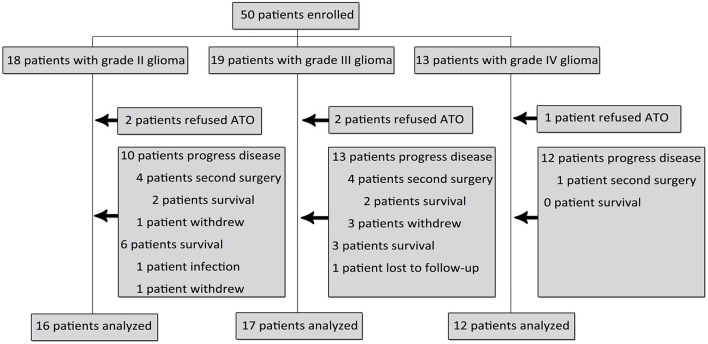
Recruitment and inclusion of glioma patients in the study.

Fifty patients with newly diagnosed glioma (WHO grade 2, 3 and 4) were recruited and enrolled (Figure 1). The main inclusion criteria were age from ≥ 18 to ≤ 70 years; Eastern Cooperative Oncology Group (ECOG) performance status ≤ 2 (29); the interval between the onset of symptoms and diagnosis in patients with glioma <2 weeks; adequate hematological function (absolute neutrophil count ≥ 1,500/ul, platelet count ≥ 100,000/ul), renal function (serum creatinine ≤ 1.5 times the upper limit of the reference range), and hepatic function (total serum bilirubin ≤ 1.5 times the upper limit of the reference range, serum transaminases ≤ 2.5 times the upper limit of the reference range). The exclusion criteria were prior RT, chemotherapy, a previous history of malignancy at other sites, or other severe underlying diseases. Female patients were required to receive adequate contraception, and pregnant women were excluded from the study.

### Treatment

All patients received partial or complete tumor resection. Two neuropathologists confirmed the intraoperative neuropathological diagnosis of glioma using frozen tumor sections. Then, the neurosurgeon completed intraoperative implantation of the Ommaya reservoir (Medtronic Inc., Goleta, CA, USA) subcutaneously. All eligible patients had a histological diagnosis of glioma according to the WHO glioma classification. In all patients, the tip of the catheter of the Ommaya reservoir was placed strategically into the tumor cavity created by surgery without involvement of the cerebral ventricles or subarachnoid space. Within six weeks after intraoperative and histological diagnosis of glioma, we assigned eligible patients to receive standard RT. Fractionated, three-dimensional conformal RT was delivered in daily fractions of 2 Gy, 5 days per week, for a total of 60 Gy. ATO was administered 5 days prior to RT and given for 5 consecutive days every 3 months for a total of eight cycles unless there was tumor progression or development of excessive toxicity. Before each session of local delivery of ATO, an intravenous injection of 250 ml of 20% mannitol incorporated with 10 mg of dexamethasone was given within 30 min. Anticonvulsants were also included. A 5-min continuous local infusion of ATO was given into the tumor cavity *via* the Ommaya reservoir using a pump.

### Patient assessment

Baseline examination was performed within 14 days before surgery, including contrast-enhanced magnetic resonance imaging (MRI), electrocardiogram, physical examination, complete medical history, full blood counts and blood chemistry tests and determination of performance status. All these tests were repeated before each course of chemotherapy, and complete blood counts were checked after each course. Postoperative contrast-enhanced MRI was used as a new baseline study for follow-up MRI comparisons that were performed before every cycle of ATO.

### Statistical analysis

The primary end point was determination of the MTD. The secondary end points were OS and PFS. Toxicity was graded according to the Common Terminology Criteria for Adverse Events version 3.0, and DLT was defined as grade 3 or 4 toxicity. Safety, toxicity and survival analyses were reported in all treated patients (intent-to-treat population) who received at least one dose of ATO. Tumor progression was defined as an increase in tumor size by 25% using the MacDonald criteria (22). OS was defined as the time from surgery until death or loss to follow-up, and PFS was calculated from the time of surgery until tumor progression or loss to follow-up according to the Kaplan-Meier method with SPSS statistical software 19.0. The 95% confidence intervals (CIs) were calculated for the survival rate. Comparisons of survival between any two grades of patients were evaluated using the log-rank test.

## Results

### Patient characteristics and treatment

Fifty patients were enrolled and implanted with Ommaya reservoirs. Five patients were excluded because of refusal of treatment. Enrollment status is summarized in Figure 1, and patient characteristics are characterized in Table 1. Histology was confirmed as WHO grade 2 glioma in 16 patients, WHO grade 3 glioma in 17 patients and WHO grade 4 glioma in 12 patients. The median age of patients in each group was 40.4, 42.6 and 45.0 years, respectively. Twenty-eight percent of patients were ≥ 50 years old. Thirty-seven patients (82%) had an ECOG performance status ≤ 1. All patients underwent craniotomy and resection of their glioma, with two-thirds of the patients having received macroscopically gross total resections as determined by neurosurgeons.

**Table 1:** Patient baseline characteristics and treatment details.

**Characteristic**	**WHO classification of glioma (*****n*** = **45)**		
	**Grade 2 (%)**	**Grade 3 (%)**	**Grade 4 (%)**
	**(*n* = 16, Trial I = 7)**	**(*n* = 17, Trial I = 5)**	**(*n* = 12, Trial I = 0)**
**Age, years**			
Median	40.4	42.6	45.0
Range	28–57	18–64	18–66
**ECOG performance**			
0	10 (63)	7 (42)	9 (75)
1	5 (31)	5 (29)	1 (17)
2	1 (6)	5 (29)	2 (8)
**Extent of surgery**			
Complete resection	12 (75)	13 (76)	5 (42)
Partial resection	4 (25)	4 (24)	7 (58)
**WHO classification of glioma**			
Fibrillary astrocytoma	3		
Oligoastrocytoma	4		
Oligodendroglioma	3		
Protoplasmic astrocytoma	1		
Astrocytoma	5		
Anaplastic astrocytoma		10	
Anaplastic ependymoma		6	
Anaplastic oligoastrocytoma		1	
Glioblastoma			12

Two patients did not complete their RT courses because of grade 4 refractory leukocytopenia; thus, their chemotherapy was also delayed. The median age of the 12 patients enrolled in the phase I trial was 43.5 years. Seven patients with WHO grade 2 glioma and five patients with WHO grade 3 glioma were enrolled in a dose escalation study according to the sequence of admission to the hospital. Dose escalation, drug cycles, acute toxicity and characteristics of the patients are shown in Table 2. All 12 patients received ATO at a dose of 2.0 mg, and ATO was discontinued in 5 patients at this dose level because of local, adverse neurological events (one patient with a grade 3 seizure; one patient with grade 3 dizziness accompanied by grade 2 vomiting lasting 3 h; three patients with grade 3 headache associated with slight drowsiness lasting <4 h). No hematological toxicity or grade 4 non-hematological toxicity was observed in patients treated with ATO during this trial. The dose recommended in the phase II trial is 1.5 mg. The patients in the phase I trial continuously received 49 cycles of ATO at a dose of 1.5 mg in the following phase II trial.

**Table 2:** Dose administered and grade 3 acute toxicity occurring in phase I study.

**Patient**	**Age**	**Diagnosis**	**Times of dose (mg)**						**Cycles**	**Grade 3 acute toxicity**
**No**.	**years**		**0.3**	**0.6**	**1.0**	**1.5**	**2.0**	**2.0**		**At a dose of 2.0 mg**
1	52	Fibrillary astrocytoma	2	1	1	1		1	7	
2	31	Anaplastic astrocytoma	2		2		1		5	Seizure
3	57	Fibrillary astrocytoma	1		1	1	1		8	
4	50	Protoplasmic astrocytoma		1	1	1		1	8	
5	42	Anaplastic oligoastrocytoma		1	1	1		1	8	
6	44	Anaplastic astrocytoma			1		1		8	Headache
7	49	Anaplastic oligodendroglioma				1		1	8	
8	51	Diffuse astrocytoma				1		1	3	Dizziness, vomiting
9	49	Anaplastic astrocytoma					2		4	
10	32	Pleomorphic xanthoastrocytoma					1		8	Headache
11	34	Oligoastrocytoma					1		8	
12	31	Oligoastrocytoma						1	8	Headache

Thirty-three patients (WHO grade 2 in 9 patients; WHO grade 3 in 12 patients; WHO grade 4 in 12 patients) who were enrolled in the phase II trial received adjuvant ATO at a dose of 1.5 mg. This dose was determined by the MTD and was informed by the phase I study. Adjuvant ATO was administered to a total of 45 patients for a total of 256 cycles. The median number of chemotherapeutic cycles per patient was 5.7. ATO was discontinued in 28 patients (62.2%) because 23 of 28 patients experienced glioma progression (10 patients with GBM; 8 patients with WHO grade 3 glioma; 5 patients with WHO grade 2 glioma). Unfortunately, the Ommaya reservoir catheters were obstructed in 2 patients, the plastic dome of the Ommaya reservoir leaked in 1 patient, and 2 patients refused to receive ATO because its toxicity resulted in headache and seizure.

### Toxicity of ATO treatment

There were no hematological toxicities or grade 4 non-hematological toxicities during treatment with ATO. Acute toxicity occurred in 18 patients when ATO was given at a dose of 1.5 or 2.0 mg. Most symptoms that these patients complained of were mild to moderate, and there were no additional toxicities observed on follow-up (Table 3). The most common symptom was headache (22.2%). The Ommaya reservoir was removed in 1 patient due to wound infection at the surgical site. At a dose of 2 mg, 3 patients had grade 3 headache, 1 patient had grade 3 dizziness associated with mild nausea, and 1 patient had mild sensation disorders lasting 2 h. At a dose of 1.5 mg, 1 patient had grade 3 headache associated with grade 2 dizziness and nausea; 6 patients had grade 1 to 2 headache, which was associated with nausea in 1 of them; 2 patients complained of paresthesia; 1 patient complained of a movement disorder (WHO grade 2) associated with expressive dysphasia but not impacting the ability to communicate, and symptoms lasted <5 h. Two patients experienced scalp burning and pain because catheter obstruction resulted in mild leakage of ATO, one patient developed alopecia surrounding the Ommaya reservoir, potentially due to leakage of ATO without catheter obstruction.

**Table 3:** Acute toxicity in trial I and II study with ATO.

**Adverse Events**	**Trial I (*****n*** = **12)**		**Trial II (*****n*** = **33)**		**(%)**
	**Grade 1/2**	**Grade 3**	**Grade 1/2**	**Grade 3**	
Headache	1 (1.5 mg)	3 (2.0 mg)	5	1	10/45 (22.2)
Dizziness		1 (2.0 mg)	1		2/45 (4.4)
Seizure		1 (2.0 mg)			1/45 (2.2)
Nausea/vomiting	1 (2.0 mg)		2		3/45 (6.7)
Local alopecia			1		1/45 (2.2)
Injection site reaction	1 (1.5 mg)		2		3/45 (6.7)
Ommaya–related infection		1			1/45 (2.2)
Paresthesia	1 (2.0 mg)		2		3/45 (6.7)
Hemiplegia			1		1/45 (2.2)
Dysphasia			1		1/45 (2.2)

### Long-term survival of GBM patients after ATO chemotherapy

**Figure 2:**
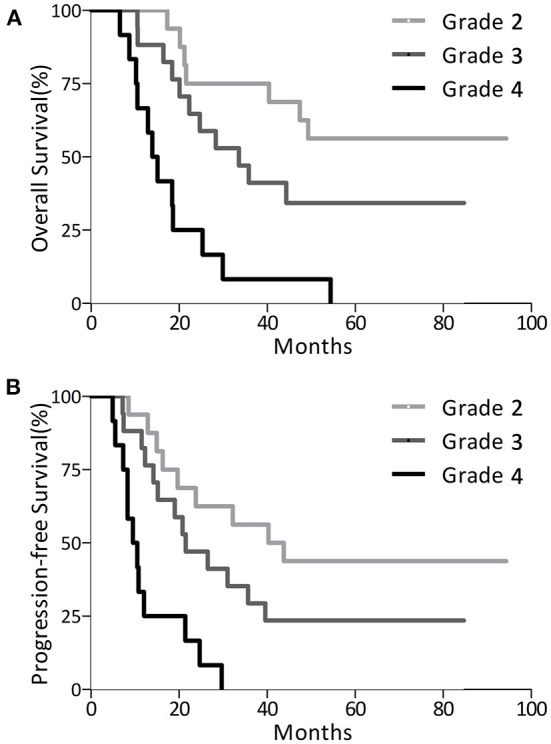
Survival analysis of glioma patients with WHO grade 2 (*n* = 16), grade 3 (*n* = 17) and grade 4 (*n* = 12). **(A)** Overall survival analysis curve. **(B)** Progression-free survival analysis curve.

In the entire cohort of 45 patients, 9 patients with WHO grade 2 glioma and 5 patients with WHO grade 3 glioma were still alive at the end of follow-up, and 1 patient with WHO grade 3 glioma was lost to follow-up. The mean duration of the follow-up was 39 months, with a maximum follow-up for surviving patients of 94.3 months. The median OS for WHO grade 2 glioma was not assessable because the number of surviving patients was over one half after 5 years; for WHO grade 3 glioma the OS was 33.6 months (95% CI, 18.7 to 48.5), and for GBM the OS was 13.9 months (95% CI, 10.2 to 17.6). The median PFS for WHO grade 2 glioma was 40.3 months (95% CI, 17.6 to 63.0), for WHO grade 3 glioma it was 21.5 months (95% CI, 11.4 to 31.6) and for GBM it was 9.5 months (95% CI, 5.8 to 13.2). The postoperative 1-5 year OS and PFS rates are listed in Table 4. Survival curves in patients with different grades of glioma are also displayed in Figure 2. The recurrence rate was greatest in the second year postoperatively compared with that in other years for WHO grade 2 or 3 glioma (the second-year progression rate was 31.2% for WHO grade 2 glioma and 35.3% for WHO grade 3 glioma). The majority (75%) of patients with GBM experienced glioma progression within 1 year, most of whom (41.6%) died within the second year.

**Table 4:** Overall survival, progression–free survival of patients with ATO treatment.

**Survival**	**WHO Grade 2**	**WHO Grade 3**	**WHO Grade 4**
		***Value (95% CI)***	
OS, months			
Median	NA	33.6 (18.7–48.5)	13.9 (10.2–17.6)
1 year (%)	100.0 (NA)	88.2 (72.9–100.0)	66.7 (40.0–93.4)
2 years (%)	75.0 (53.8–96.2)	64.7 (41.9–87.4)	25.0 (0.5–49.5)
3 years (%)	75.0 (53.8–96.2)	41.2 (17.9–64.5)	8.3 (0.0–23.7)
4 years (%)	62.5 (38.8–86.2)	34.3 (11.2–57.4)	8.3 (0.0–23.7)
5 years (%)	56.3 (32.0–80.6)	34.3 (11.2–57.4)	0.0
PFS, months			
Median	40.3 (17.6–63.0)	21.5 (11.4–31.6)	9.5 (5.8–13.2)
1 year (%)	93.8 (81.8–100.0)	82.4 (64.4–100.0)	25.0 (0.5–49.5)
2 years (%)	62.5 (38.8–86.2)	47.1 (23.4–70.8)	16.7 (0.0–37.9)
3 years (%)	56.3 (32.0–80.6)	29.4 (7.7–51.2)	0.0
4 years (%)	43.8 (19.5–68.1)	23.5 (3.3–34.7)	0.0
5 years (%)	43.8 (19.5–68.1)	23.5 (3.3–34.7)	0.0

NA, not assessable.

## Discussion

Malignant glioma is notorious for its progression and recurrence. Various treatment strategies for glioma have been applied to improve treatment outcomes, including the combination of TMZ with other commonly used chemotherapeutic reagents such as lomustine or vincristine (7, 8). However, as individual agents, these have never been conclusively demonstrated to have a clear clinical benefit (7, 9, 30). Moreover, various degrees of systemic toxicities from these agents have been reported (31, 32). The concept that chemotherapeutic agents can be locally administered through an Ommaya reservoir has been proposed to improve antitumor efficacy by bypassing the BBB with reduced systemic toxicity (26, 33). Several studies demonstrated that ATO can repress glioma growth through several mechanisms (21, 22, 34–36). Phase I studies of ATO systemically administered in adult and pediatric patients with glioma reported promising effects (24, 25). A higher local drug concentration and better antitumor efficacy may be demonstrated if ATO is administered directly into the tumor cavity. Safety and tolerance issues of ATO delivered into the brain in this manner have not yet been reported. Our aim was to evaluate the safety and efficacy of ATO as a single adjuvant chemotherapeutic reagent administered locally in patients with different grades of glioma.

The initial dose of ATO was 0.3 mg, based on findings from a previous animal study in which dose-limiting toxicity was reached at doses up to 10 μg/d in mice (27). The total course of chemotherapy lasts for almost 6 months in the majority of cases (4, 7, 9). However, GBM often recurs 7 months after surgery and patients with low-grade glioma have a longer interval from tumor resection to recurrence (9). This phenomenon suggests that the increase in the interval between cycles may be an effective strategy for the treatment of glioma. After a 3-month break, patients received up to 8 cycles of ATO according to the standard 5-day schedule every 3 months. This strategy may prolong periods of disease stabilization or postpone recurrence of glioma. A phase I dose escalation study of ATO was performed in every patient because a 3-month interval would eliminate the impact of dose escalation on the assessments of both MTD and ineffective dosing in the same patients. Our results in this trial demonstrated that adjuvant ATO may result in mild to moderate non-hematological toxicities. In addition, no hemorrhagic or severe non-hematological complications were observed in this population of patients treated with ATO. Therefore, the use of ATO is safe and well tolerated at a dose of 1.5 mg. Headache was often observed and may be due to local irritation of the meninges. There were no additional toxicities reported at follow-up periods. A patient whose weight is 75 kg needs to receive a total daily dose of 15 mg if ATO is systemically administered at a dose level of 0.2 mg/kg (24). The dose of 1.5 mg in the tumor cavity is relatively higher than the drug concentration in the brain after 15 mg of ATO is administered systemically.

ATO has shown promising efficacy in patients with WHO grade 2/3/4 glioma. Our results demonstrated that a better clinical outcome was observed in patients with GBM with a median OS of 13.9 months. While the survival benefit may be attributed to the efficacy of ATO, there are some limitations that need to be considered. These limitations include the fact that most patients were not willing to receive further therapy after their tumors progressed or relapsed. Only 8 of 33 patients (24%) with WHO grade 2 or 3 glioma were treated with second-time therapy after tumor recurrence, and 4 of them were still alive. The relatively small cohort might have diminished the statistical power of this study. Another limitation of this study is that we did not administer ATO in combination with TMZ, which has demonstrated clinical efficacy (24), because TMZ is an expensive therapy for patients with glioma in China. Before clinical application, further studies are required to improve and develop the method of ATO local interstitial chemotherapy, to explore the safety and efficacy for patients with recurrent glioma and to investigate of the therapeutic effects of ATO combined with TMZ for glioma.

## Conclusion

In conclusion, for the first time, we have demonstrated that ATO is safe and well tolerated when locally delivered into a glioma, and the dose recommended for a phase II trial is 1.5 mg. ATO has shown potential for local chemotherapy to treat patients with newly diagnosed glioma. A study to explore the further chemotherapeutic effects of ATO on glioma is currently under investigation in our laboratory.

## Data availability statement

The original contributions presented in the study are included in the article/supplementary material, further inquiries can be directed to the corresponding author.

## Ethics statement

The studies involving human participants were reviewed and approved by Ethics Committees of the First Affiliated Hospital of Harbin Medical University. The patients/participants provided their written informed consent to participate in this study. Written informed consent was obtained from the individual(s) for the publication of any potentially identifiable images or data included in this article.

## Author contributions

DH, LT, and XW contributed equally to this manuscript. SZ designed and supervised this research. AS, DH, LT, XW, and YZ analyzed data. MY, MH, GY, MG, JX, and YL collected the data. AS, DH, and LT provided the administrative, technical, or material support. AS, DH, LT, XW, YZ, GY, LW, and XC wrote, reviewed, and revived this manuscript. All authors read and approved the final manuscript.

## Funding

This work was supported by the National Natural Science Foundation of China (81272788 and 81472368, SZ; 81402062, DH; 81902554, XW), Grant-in-Aid for Scientific Research of Heilongjiang Province (2013184, DH), Fund of Science Research, Education Department of Heilongjiang Province (1254HQ006, LT), Fund for returned researchers, Department of International Cooperation and Communication, Ministry of Education of the People's Republic of China (No. 49, LT), Scientific Research and Innovation Foundation of the First Affiliated Hospital of Harbin Medical University (2021M05, DH), and the First Affiliated Hospital of Harbin Medical University Foundation (2013Lx03, LT).

## Conflict of interest

The authors declare that the research was conducted in the absence of any commercial or financial relationships that could be construed as a potential conflict of interest.

## Publisher's note

All claims expressed in this article are solely those of the authors and do not necessarily represent those of their affiliated organizations, or those of the publisher, the editors and the reviewers. Any product that may be evaluated in this article, or claim that may be made by its manufacturer, is not guaranteed or endorsed by the publisher.

## Abbreviations

ATO: Arsenic trioxide
MTD: Maximum tolerated dose
GBM: Glioblastoma
TMZ: Temozolomide
BBB: Blood-brain barrie
RT: Radiotherapy
OS: Overall survival
PFS: Progression-free survival
DLT: Dose-limiting toxicity
ECOG: Eastern Cooperative Oncology Group
MRI: Magnetic resonance imaging
CIs: Confidence intervals.
